# Prophylactic Sclerotherapy: Yes or No!

**DOI:** 10.1155/1996/38241

**Published:** 1996

**Authors:** A. K. Burroughs

**Affiliations:** Consultant Physician & Hepatologist University Department of Medicine Royal Free Hospital and Department of Medicine Pond Street Hampstead London NW3 2QG United Kingdom

## Abstract

Controlled trials of endoscopic sclerotherapy for the prevention of the first variceal hemorrhage have given controversial results. We continued a previously reported study and randomly assigned 141 patients with esophageal varics and no prior gastrointestinal bleeding to either prophylactic sclerotherapy (*n*=70) or no treatment (*n*=71). Sclerotherapy was performed until complete eradication of the varices was achieved; recurrent varics were treated with repeat sclerotherapy. The groups were well balanced in terms of demographic and clinical characteristics. Patients in both groups who bled from varices received sclerotherapy whenever possible.

During a median follow-up of 56 months, variceal bleeding occurred in 7% in sclerotherapy patients and 44% on control patients (*p* < 0.01). In the sclerotherapy group 59% died, and in the control group 51% (n.s.). In both groups, the mortality rate increased with the severity of liver function impairment. Sclerotherapy was not found to improve survival in patients, irrespective of the etiology of cirrhosis (alcoholic or nonalcoholic) or variceal size (low-grade or high-grade). We conclude that sclerotherapy is a suitable method to reduce the occurrence of the first variceal hemorrhage, but it does not appear to have an effect on survival.


*HPB Surgery*, 1996, Vol. 9, pp.185–189
Reprints available directly from the publisher
Photocopying permitted by license only

© 1996 OPA (Overseas Publishers Association)
Amsterdam B.V. Published in The Netherlands
by Harwood Academic Publishers GmbH
Printed in Malaysia

### HPB INTERNATIONAL

**EDITORIAL & ABSTRACTING SERVICE**

JOHN TERBLANCHE EDITOR

Department of Surgery,
Medical School · Observatory 7925
Cape Town · South Africa
Telephone: 27-21-406-6204
Telefax 27-21-448-6461
E-mail: JTER BLAN@UCT GSHI.UCT.AC.2A

### PROPHYLACTIC SCLEROTHERAPY: YES OR NO!

### ABSTRACT

*Koch, H., Binmoeller, K.F., Grimm, H., Soehendra, N., Henning, H. and Oehler, G. (1994) Prophylactic sclerotherapy for esophageal varices: Long-term results of a prospective study. Endoscopy; 26: 729–733.*

**Controlled trials of endoscopic sclerotherapy for the prevention of the first variceal hemorrhage have given controversial results. We continued a previously reported study and randomly assigned 141 patients with esophageal varics and no prior gastrointestinal bleeding to either prophylactic sclerotherapy ($n = 70$) or no treatment ($n = 71$). Sclerotherapy was performed until complete eradication of the varices was achieved; recurrent varics were treated with repeat sclerotherapy. The groups were well balanced in terms of demographic and clinical characteristics. Patients in both groups who bled from varices received sclerotherapy whenever possible.**

**During a median follow-up of 56 months, variceal bleeding occurred in 7% in sclerotherapy patients and 44% on control patients ($p < 0.01$). In the sclerotherapy group 59% died, and in the control group 51% (n.s.). In both groups, the mortality rate increased with the severity of liver function impairment. Sclerotherapy was not found to improve survival in patients, irrespective of the etiology of cirrhosis (alcoholic or nonalcoholic) or variceal size (low-grade or high-grade). We conclude that sclerotherapy is a suitable method to reduce the occurrence of the first variceal hemorrhage, but it does not appear to have an effect on survival.**

*Paquet, K-J., Kalk, J-F, Klein, C-P. and Gad, H.A. (1994) Prophylactic sclerotherapy for esophageal varices in high-risk cirrhotic patients selected by endoscopic and hemodynamic criteria: A randomised, single-center controlled trial. Endoscopy; 26: 734–740.*

**Controlled trials of sclerotherapy for the prevention of the first variceal hemorrhage in cirrhotics have given conflicting results, in spite of an initial positive controlled trial. We designed therefore a new study in which only 89 of 396 investigated patients were randomized to sclerotherapy (44 patients) or a control group (45 patients). The admis-**

**sion criteria were: no history of variceal bleeding, the presence of high risk varices, i.e, varcies of degrees III and IV with minivarices on the surface of them, and portal pressure over 16 mmHg. Sclerotherapy sessions were performed at 0, 7, 14, 21, and 28 days, untill the varices were reduced in size and completely covered by fibrous tissue. Follow-up endoscopy was performed at four-month and thereafter at six-month intervals. The control patients underwent repeated clinical investigation and endoscopy at six-month intervals. Bleeding episodes were treated by emergency endoscopic sclerotherapy in both groups, whenever possible. The mean follow-up was 33 months. The results were analyzed using Student's *t*-test and the log-rank test. Variceal bleeding occured in 11 sclerotherapy patients (25%) and 34 controls (75.6%) ($p < 0.05$). The overall mortality was 25% (11 patients) among the sclerotherapy patients and 69% (31 patients) in the controls ($p < 0.01$). Prophylactic endoscopic sclerotherapy was able to prolong survival in Child-Pugh classes A and B, but not in C. It is concluded that prophylactic endoscopic sclerotherapy does reduce the incidence of first variceal bleeding in cirrhotic patients, and is able to prolong survival if only high-risk patients are selected and the treatment is performed by endoscopic experts.**

KEYWORDS: Scleotherapy Prophylactic sclerotherapy oesophageal varices.

### PAPER DISCUSSION

The benefits of prophylactic sclerotherapy in cirrhotics who have not bled from varices are controversial. As beta blockers have been shown to have benefit (1) with clinical and statistical homogeneity as regards efficacy, beta blockers are the current standard and accepted therapy. A meta-analysis of beta blocker therapy, propranolol or nadolol, reduced the incidence of bleeding at a statistically significant level and increase survival albeit, this just misses statistical significance (1).

The results of sclerotherapy have been very varied, meta-analysis showing heterogeneity i.e. a wider variation than that expected by chance considering each trial as a repeated experiment (1). The reasons for heterogeneity might lie in different operators e.g. the American studies report a higher frequency of serious side-effects and have increased mortality (2) or in different populations–alcoholics may respond differently (3). However, the most obvious reason lies in the different baseline bleeding risks of the populations which entered these studies, from 20% to 70%. The high baseline risk of bleeding, either means there has been selection of patients, but this is not apparent from the studies, or mis-selection perhaps including patients who had had minor bleeds. Interestingly there is a relationship between greater efficacy of sclerotherapy and lower quality score for each trial when reasons for heterogeneity were examined (1).

It is now known that large varices, red signs and worse liver function predict a higher bleeding risk (4), so that selection of patients might be possible. Lastly, it should be remembered that sclerotherapy does not prevent bleeding from portal hypertensive gastropathy, and this is a source of first gastrointestinal bleeding in cirrhotics.

Against this background one can evaluate the results of 2 recently published prophylactic sclerotherapy trials. One by Koch *et al.* is a longer follow up report with more patients of a previously published study (5), and the second is a new trial by Paquet *et al.* the authors of the first prophylactic study (6) who repeated a prophylactic study with selection of cirrhotics at high risk of first bleeding.

The trial by Koch *et al.* describes 2 separate trials. There was an initial recruitment period which represented the first study published (5), then a gap of 3 years before further recruitment. The latter is in effect a separate study and the results should have been presented as separate. Unfortunately, the data was not presented for the second period of recruitment. A total of 140 patients were recruited. There was no selection for size of varices. Polidocanol 1% was injected intra and para variceal. The follow up was less intensive in the control group every 12 months, compared to the close initial follow up because of the variceal injections in the sclerotherapy group, and, therefore, 3–6 monthly. The mean follow up was 56 months (26 to 140 months). There were 13 of 70 patients bleeding in the sclerotherapy group compared to 31 of 70 in the control group ($p < 0.001$) and death due to bleeding was 6 in the injected group and 17 in the control group. Surprisingly no patient is documented as bleeding from portal hypertensive gastropathy–perhaps this did occur but was not evaluated. Despite this there was no difference in mortality by Kaplan-Meier plots. In fact there were more deaths in the sclerotherapy group, 41, than the control group 36. As death due to bleeding was 50% in the control group and 85% in the injected group one would have expected a difference in survival. Serious complications of sclerotherapy were low in the second period, only 1 patient, compared to 6 in the first phase.

This trial shows no survival benefit for prophylactic sclerotherapy. The benefit in prevention of bleeding was not evaluated in terms of length of hospital stay, endoscopy workload nad patient acceptability. Given that the phased recruitment may have influenced results, and that beta blockers are cheaper and simpler to administer, this trial does not change current practice for prophylaxis of variceal bleeding nor suggest further studies of sclerotherapy.

The trial by Paquet *et al.* is far more interesting. In this study a deliberate attempt was made to recruit patients at high risk of bleeding. Thus, those with large varices and "varices on varices" (red signs)(4) together with those with a hepatic venous pressure gradient of >16 mmHg were selected. From a consecutive series of 396 patients only 89 fulfilled the criteria. Patients in the control group were followed up 6 monthly so that there was an attempt to make the frequency of visits in the 2 groups similar. Polidocanol 0.5% to 1% was injected para and intravariceally. In this study there was a marked difference in the occurrence of upper gastrointestinal bleeding: 13 of 44 in the sclerotherapy group but 35 of 45 in the control group ($p < 0.05$). Portal hypertensive gastropathy bleeding was reported in 3. Survival was also improved in the sclerotherapy group 11 v 31 deaths ($p < 0.01$). When analysed by Child's groups, this was only true for groups A and B, as there was no difference in Child's C patients. In this trial there is the expected improvement in survival, secondary to the reduction in bleeding. There were 3 major complications in the sclerotherapy group (including 1 ulcer bleed included in the bleeding group above). The authors appear to have selected out a population with at least a 70% bleeding risk, the highest in the literature. Based on the North Italian Endoscopic Club criteria of a bleeding risk of at least 40%, another prophylactic sclerotherapy study (7) could not show benefit in survival, although there was a difference in bleeding.

Whether the addition of the haemodynamic parameter of hepatic venous pressure gradient allowed Paquet *et al.* to more accurately select a group at high risk of bleeding from varices remains to be proven by further study. It is feasible that this cut off is clinically significant, as for similar size varices a greater pressure will increase tension on the wall to a greater degree and cause rupture of the varix.

New prophylactic trials for prevention of first variceal bleeding should target subgroups of cirrhotics at greater risk of bleeding. Portal pressure should be measured ab initio and beta blocker therapy should be the control arm. The study by Paquet *et al.* has reopened the question of prophylactic endoscopic treatment for varices but again does not indicate that a change in current clinical practice is needed. Beta blockers remain the treatment of choice.

Whether banding ligation should replace sclerotherapy, also needs to be evaluated. The complication rate in these 2 trials is lower than previously reported with sclerotherapy. Overtube complications with banding are potentially serious, so that it may not be an automatic choice for endoscopic treatment prophylactically. In any case banding ligation should only be compared to beta-blockers. Ideally measurement of portal pressure should be undertaken, to confirm Paquet's results.

### REFERENCES

1. Pagliaro L, D'Amico G, Sorensen A, Lebrec D, Burroughs AK, Morabito A, Tine F, Politi, Traina M. (1992) Prevention of first bleeding in cirrhosis. A meta analysis of randomized trials of non-surgical treatment. *Ann Int Med* **117**: 59–70.
2. Veterans Affairs Cooperative Variceal Sclerotherapy Group. (1991) Prophylactic sclerotherapy for esophageal varices in men with alcoholic liver disease. *N Eng J Med* **324**: 1776–1784.
3. Burroughs AK, D'Heygere F, McIntyre N. (1986) Pitfalls in studies of prophylactic therapy for variceal bleeding in cirrhotics. *Hepatology* **6**: 1407–13.
4. North Italian Endoscopic Club for the Study and Treatment of Esophageal varices: (1988) Prediction of the first variceal haemorrhage in patients with cirrhosis of the liver and oesophageal varices: A prospective multicentre study. *N Engl J Med* **319**: 983–989.
5. Koch H, Henning H, Grimm H *et al.* (1986) Prophylactic sclerosing of esophageal varices: results of a prospective controlled study. *Endoscopy* **18**: 40–43.
6. Paquet K.J. (1982) Prophylactic endoscopic sclerosing treatment of the oesophageal wall in varices: a prospective controlled randomised trial. *Endoscopy* **14**: 4–5.
7. DeFranchis R, Primignani M, Archidiacono PG *et al.* (1991) Prophylactic sclerotherapy in high-risk cirrhotics selected by endoscopic criteria. *Gastroenterology* **101**: 1087–93.

Dr AK Burroughs MB ChB Hons FRCP
Consultant Physician & Hepatologist
University Department of Medicine
Royal Free Hospital and Department of Medicine
Pond Street
Hampstead
London NW3 2QG
United Kingdom

